# Tailoring Borate Mediator Species Enables Industrial CO Production with Improved Overall Energy Efficiency by Sustainable Molten Salt CO_2_ Electrolysis

**DOI:** 10.1002/advs.202406457

**Published:** 2024-12-04

**Authors:** Xinyu Li, Bowen Deng, Kaifa Du, Wenmiao Li, Di Chen, Xin Qu, Fangzhao Pang, Xiaodan Zhang, Hao Zha, Huayi Yin, Dihua Wang

**Affiliations:** ^1^ School of Resource and Environmental Science Wuhan University Wuhan 430072 P. R. China; ^2^ Hubei International Scientific and Technological Cooperation Base of Sustainable Resource and Energy Wuhan University Wuhan 430072 P. R. China; ^3^ State Key Laboratory of Water Resources Engineering and Management Wuhan University Wuhan 430072 P. R. China

**Keywords:** CO production, electrochemical CO_2_ reduction, electrode processes, molten salt electrolysis, oxygen evolution reaction, reaction kinetics

## Abstract

The electrochemical conversion of CO_2_ into CO represents a promising strategy for mitigating excessive global greenhouse gas emissions. Nevertheless, achieving industrial‐scale electrochemical CO_2_‐to‐CO conversion with enhanced selectivity and reduced energy consumption presents significant challenges. In this study, a borate‐enhanced molten salt process for CO_2_ capture and electrochemical transformation is employed, achieving over 98% selectivity for CO and over 55% energy efficiency without the necessity for complex and costly electrocatalysts. Cathodic CO_2_ electro‐reduction (CO_2_ER) with the anodic oxygen evolution reaction (OER) at an overall current density of 500 mA cm^−2^ using non‐nanostructured transition‐metal plate electrodes at 650 °C is coupled. By regulating the electrolyte's oxo‐basicity with earth‐abundant borax (Na_2_B_4_O_7_), a borate‐enhanced electrolyte is established that accelerates the overall electrochemical reaction efficiently. This system involved a series of well‐designed target borate species (BO_3_^3−^, BO_2_^−^, and B_4_O_7_^2−^) that acted as mediators shuttling between the cathode and anode, favoring CO as the primary cathodic product. Manipulating the atmosphere above the anode facilitated a spontaneous transformation of borates, further enhancing OER performance with long‐term operational stability over a cumulative period of 50 h, while also reducing overall energy consumption. This work presents a cost‐effective strategy for the industrial‐scale production of CO derived from CO_2_, contributing to a lower carbon footprint by establishing a sustainable borate‐mediated closed loop.

## Graphical abstract

This study introduces a borate‐enhanced molten salt electrochemical process for the efficient capture and conversion of CO_2_ to CO. By optimizing the electrolyte composition and coupling cathodic CO_2_ reduction with anodic oxygen evolution, the system achieves over 98% Faradaic efficiency and 55% energy efficiency at a current density of 0.5 A cm^−2^, all without the use of precious‐metal catalysts.

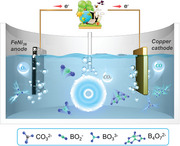

## 1 Introduction

Carbon dioxide (CO_2_) capture and utilization processes present potential solutions for addressing the rising concentration of atmospheric CO_2_,^[^ ^1^, ^2^ ^]^ such as chemically catalytic hydrogenation,^[^ ^3^ ^]^ CO_2_ photocatalytic reduction,^[^ ^4^ ^]^ CO_2_ biocatalysis,^[^ ^5^ ^]^ and CO_2_ electrochemical reduction (CO_2_ER),^[^ ^6^ ^]^ etc. Among these methods, CO_2_ER emerges as a promising approach to converting CO_2_ into valuable fuels and chemicals, offering a sustainable way to store intermittent renewable energy (e.g., solar and wind).^[^ ^7^ ^]^ Joint efforts have been concentrated on improving the efficiency and selectivity by exploring different electrolyte systems (aqueous solutions, ionic liquids, high‐temperature solid oxides, and molten salts),^[^ ^8^ ^]^ designing electrolyzer configurations (membrane electrode assembly, gas diffusion electrode),^[^ ^9^ ^]^ and developing complex nanostructured electrocatalysts (single‐atom catalysts, noble metal nano‐catalysts, and transition‐metal nano‐clusters and their nano‐composites).^[^ ^10^ ^]^ However, a cost‐effective strategy is still an urgent need.^[^ ^8^ ^]^

Among these electrolytes, molten salt electrolytes have attracted increasing attention due to their wide electrochemical window, excellent mass/heat transfer properties, and relatively high CO_2_ solubility (hundreds of moles of CO_2_ per cubic meter of salt),^[^ ^11^ ^]^ which potentially allows high‐flux CO_2_ absorption and electrochemical conversion with rapid reaction kinetics and relatively high current efficiency, called molten salt CO_2_ capture and electro‐transformation (MSCC‐ET) process.^[^ ^12^, ^13^, ^14^ ^]^ Similar to other typical CO_2_ electrolysis systems, this process conventionally involves a cathodic reaction that CO_2_, or the captured CO_2_ (in the form of carbonate ions) is electrochemically converted into advanced carbon materials (*CO*_3_^2−^ + 4*e* ^−^ = *C* + 3*O* ^2−^), CO, and carbon fuels (co‐electrolysis with water vapor), while the anodic reaction is expectedly coupled with oxygen evolution reaction (OER) originating from oxide ions (2*O* ^2−^ −4*e* ^−^ = *O*_2_(*g*)). Considering that oxide ions (O^2−^) are released at the cathode side and will serve as OER precursors and CO_2_ absorbents (*CO*_2_(*g*) + *O* ^2−^ = *CO*_3_^2−^), they are key mediators to sustain electrode reactions and keep the mass balance of the entire electrochemical process.

Compared to other products, the advanced carbonaceous materials, such as carbon nanotubes/nanofibers,^[^ ^15^, ^16^ ^]^ hollow carbon spheres,^[^ ^17^ ^]^ carbon particles,^[^ ^18^ ^]^ and graphite as well as their derivate composites,^[^ ^19^, ^20^ ^]^ are thermodynamically preferential cathodic products for MSCC‐ET over a wide temperature range of 400–900 °C,^[^ ^12^ ^]^ of which nanostructures and morphology can be regulated by applying suitable electrolysis parameters (operating temperature, electrolyte component, and electrode materials). Due to their unique nanostructures/crystallinity, tunable specific surface area, and rich oxygen‐containing functional group, the advanced carbonaceous materials have been widely used as energy storage materials (e.g., Li‐ion, Na‐ion battery negative electrodes, and supercapacitors),^[^ ^21^, ^22^, ^23^ ^]^ contaminant adsorbents/degradation catalysts,^[^ ^23^, ^24^ ^]^ and heat storage additives,^[^ ^25^ ^]^ so forth. In stark contrast to carbon products, CO, an important building block for the modern chemical industry, is thermodynamically favored either at a high operating temperature(e.g., 900 °C) or in the dependence of electrode configuration/materials alternatively. For example, CO can be achieved at ≈100% current efficiency no more than 100 mA cm^−2^ in molten Li_2_CO_3_ with the dependence of a Ti cathode at 900 °C.^[^ ^26^ ^]^ A mixed product of C and CO was observed in CaCl_2_‐CaO at 900 °C.^[^ ^27^ ^]^ By using a deliberately designed porous Fe foam electrode to support CO_2_ direct electroreduction, CO is the primary product reaching over 90% current efficiency at 100 mA cm^−2^ at 750 °C,^[^ ^28^ ^]^ however, it is still challenging to achieve an industrial‐scale CO_2_‐to‐CO conversion (e.g., 500 mA cm^−2^) under mild conditions (e.g., ≤650 °C). So far, many efforts have been made to regulate cathodic reaction as well as the product selectivity, unfortunately, few attentions are focused on the anode side, while the anodic reaction pathway will also determine the applied cell voltage as well as the net CO_2_ conversion efficiency. To be specific, due to the limited solubility of oxide ions, the carbonate‐ions‐involved OER reaction (2*CO*_3_^2−^ −4*e* ^−^ = 2*CO*_2_ + *O*_2_), instead of the oxide‐ions‐involved one, will eventually be a dominant anodic, particularly under a strong electrode polarization condition (e.g., a high current density),^[^ ^29^ ^]^ where a mixture of CO_2_ and O_2_, rather than O_2_ only, will be generated at the anode, decreasing the net CO_2_ conversion efficiency, because carbonates and carbonate‐containing melts are the mainstream electrolyte candidates for molten salt CO_2_ electrolysis.^[^ ^30^ ^]^

To address the aforementioned bottlenecks, we selected cost‐effective and earth‐abundant borax (Na_2_B_4_O_7_) as an electrolyte reactant to naturally generate tailored borate species under manually regulated conditions.^31^ This selection not only favored CO as the primary product of CO_2_ER at the cathode but also enhanced oxygen evolution reaction (OER) with pure oxygen (O_2_) generation at the anode. The tailored borates contributed to a significant enhancement in the kinetics of both electrode reactions, allowing for operation under considerably reduced polarization conditions. The influences of Na_2_B_4_O_7_ content, operating temperature, and atmosphere on industrial‐scale CO_2_‐to‐CO conversion were systematically investigated. Moreover, the long‐term stability of borate‐enhanced molten salt CO_2_ electrolysis was also revealed.

## 2 Results and Discussion

### 2.1 Thermodynamic Analysis of the Cathode Process

**Figure 1:**
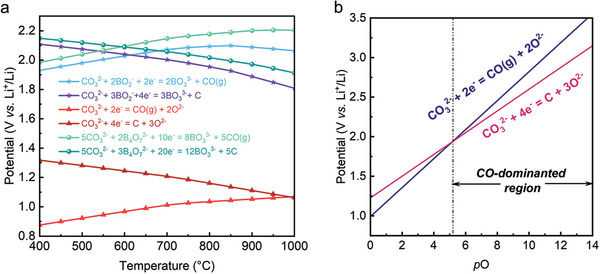
a) The theoretical potential of possible cathodic reactions at different operating temperatures. b) The correlation between the activity of oxide ion and cathodic potential, $pO=-lga_{O^{2-}}$. The thermodynamic data is sourced from HSC 9.0 software database.

Raising the operating temperature is beneficial to overcome the required energy barrier (Figure S1c, Supporting Information). In the traditional understanding, MSCC‐ET technology features a continuous CO_2_ capture and electrochemical conversion loop.^[^ ^32^ ^]^ Initially, CO_2_ is captured by oxide ions (O^2−^) in the electrolyte, forming carbonate ions (CO_3_^2−^), which then migrate to the cathode for electrochemical production of C or CO, releasing O^2−^. Some of the released O^2−^ continues the cycle by capturing CO_2_ to regenerate CO_3_^2−^ (the CO_2_ER electrolytic precursor), while the remaining O^2−^ migrates to the anode for O_2_ generation. Molten Li_2_CO_3_‐Na_2_CO_3_‐K_2_CO_3_ (43.5: 31.5: 25 in molar ratio, referred to LNK) is a typical molten salt CO_2_ electrolysis electrolyte due to its low melting point, high CO_2_ absorption capacity, and rapid mass transfer. Taking LNK as an example, thermodynamic analysis of the cathode process of CO_2_ER is illustrated in **Figure** **1**a. The analysis reveals that carbon is the primary cathodic product over a wide temperature range below 900 °C, as the electroreduction potential for CO_2_‐to‐C conversion is more thermodynamically favorable compared to CO_2_‐to‐CO conversion. These findings suggest that achieving CO production at relatively lower temperatures is challenging unless the thermodynamic pathway of the reaction is altered.

Traditional cathodic reaction pathway:

(1) $$\begin{array}{ccc} {\mathrm{CO}}_{3}^{2-}+4e^{-} & = & C+3O^{2-}\;\;E=E^{0}-\frac{3\cdot 2.3\mathrm{RT}}{nF}lg\;a_{O^{2-}}\\ E^{0} & = & 1.27\;V\;versus\;Li^{+}/Li \end{array}$$

(2) $$\begin{array}{ccc} {\mathrm{CO}}_{3}^{2-}+2e^{-} & = & \mathrm{CO}+2O^{2-}\;\;E=E^{0}-\frac{2\cdot 2.3\mathrm{RT}}{nF}\mathrm{lg}\;a_{O^{2-}}\\ E^{0} & = & 0.99\;V\;\mathrm{vers}\mathrm{us}\;Li^{+}/\mathrm{Li} \end{array}$$

Besides, due to the limited solubility of oxide ions in most molten salt electrolytes and sluggish diffusion kinetics, the accumulation of oxide ions (O^2−^) released during CO_2_ electrolysis on the cathode side can lead to their precipitation as solid oxides, such as Li_2_O solids. This can alter cathode viscosity and cause passivation effects.^[^ ^33^ ^]^ Additionally, it reduces oxide ion content in the electrolytes, impacting CO_2_ capture efficiency. This issue is exacerbated under high current density conditions, leading to increased electrode polarization and reduced energy efficiency in CO_2_ electrolysis. Based on these findings, we correlate the activity ($a_{O^{2-}}$) of O^2−^with the potential of CO_2_ER according to Nernst equation (Equations 1 and 2). As shown in Figure 1b, surprisingly, thermodynamic results show that changes in oxide ion activity have been found to affect the selectivity of CO_2_ electroreduction products. To be specific, when O^2−^ activity is low, the product selectivity shifts from carbon to CO, suggesting that reducing O^2−^ activity could convert CO_2_ to CO under milder conditions.

Oxide ions are bases with a high affinity for absorbing CO_2_. According to the Lux‐Flood acid‐base theory, chemicals capable of accepting O^2−^ are referred to as oxo‐acids, while those able to donate O^2−^ are classified as oxo‐bases.^[^ ^34^ ^]^ Introducing oxo‐acidic groups that can react spontaneously with oxide ions may convert them into other oxo‐basic groups, allowing for the capture of CO_2_ while maintaining their inherent properties. Among different promising candidates, the earth‐abundant borax (Na_2_B_4_O_7_) as well as its derivatives are a class of cost‐effective, inexpensive, and potentially viable additives due to their wide range of oxo‐acidity. Their corresponding reactions with oxide ions are displayed as follows:^[^ ^35^ ^]^

Transformation of O^2−^:

(3) $$B_{4}O_{7}^{2-}+O^{2-}\;⇌\;4BO_{2}^{-}\;\;\;\;\;\;\;\Delta_{r}G=-136.2\;kJ\;mol^{-1}$$

(4) $$BO_{2}^{-}+O^{2-}⇌\;BO_{3}^{3-}\;\;\;\;\;\Delta_{r}G=-102.3\;kJ\;mol^{-1}$$

Overall reaction:

(5) $$\;B_{4}O_{7}^{2-}+5O^{2-}⇌\;4BO_{3}^{3-}\;\;\;\;\;\Delta_{r}G=-545.4\;\mathrm{kJ}\;\mathrm{mo}l^{-1}$$

CO_2_ capture by oxo‐basic borate species:

(6) $$BO_{3}^{3-}+CO_{2}⇌\;CO_{3}^{2-}+BO_{2}^{-}\;\;\;\;\;\;\Delta_{r}G=-43.4\;kJ\;mol^{-1}$$

Combining Equations (1) and (2) with Equations (4) and (5), then, the borate‐assisted cathodic reactions are displayed as follows:

(7) $$\begin{array}{ccc} & & {\mathrm{CO}}_{3}^{2-}+4e^{-}+3/5B_{4}O_{7}^{2-}\\ & & \;=C+12/5{\mathrm{BO}}_{3}^{3-}\;\;E^{0}=2.07\;V\;versus\;Li^{+}/Li \end{array}$$

(8) $${\mathrm{CO}}_{3}^{2-}+4e^{-}+3{\mathrm{BO}}_{2}^{-}=C+3{\mathrm{BO}}_{3}^{3-}\;\;E^{0}=2.02\;V\;versus\;Li^{+}/Li$$

(9) $$\begin{array}{ccc} & & C{O_{3}}^{2-}+2e^{-}+2/5B_{4}{O_{7}}^{2-}\\ & & \;=\mathrm{CO}+8/5B{O_{3}}^{3-}\;\;E^{0}=2.12\;V\;versus\;Li^{+}/Li \end{array}$$

(10) $$\begin{array}{c} C{O_{3}}^{2-}+2e^{-}+2{\mathrm{BO}}_{2}^{-}=\mathrm{CO}+2B{O_{3}}^{3-}\;\;E^{0}=2.05\;V\;versus\;Li^{+}/Li\;\;\;\; \end{array}$$

The thermodynamics of the CO_2_ER reaction involving borate is also illustrated in Figure 1a. It is observed that the presence of borates leads to a significant positive shift of at least 800 mV in the theoretical potentials for CO_2_‐to‐C and CO_2_‐to‐CO compared to borate‐free conditions. This indicates that CO_2_ER can be initiated under lower cathode polarization conditions. Furthermore, the study demonstrates that the selectivity of CO_2_ER products can be altered at a lower operating temperature. In contrast to traditional electrolyte systems where achieving CO_2_‐to‐CO conversion requires temperatures exceeding 900 °C, the borate‐enhanced thermodynamic pathway suggests that CO can be obtained at temperatures as low as 600 °C. Notably, B_4_O_7_^2−^ plays a more prominent role in the positive shift of CO_2_ER potential compared to BO_2_^−^, likely due to varying reaction tendencies of different oxo‐acidic borate forms toward oxide ions. Higher reactivity of oxo‐acidic borates with oxide ions results in a greater change in Gibbs free energy (Equations 4 and 5), leading to reduced cathodic polarization. Thus, the study speculates that manipulating the form of oxo‐acidic borate can significantly decrease cathodic polarization during cathode processes. As a result, borax was chosen as an electrolyte additive in this work, and a borate buffer system comprising B_4_O_7_^2−^, BO_2_^−^, and BO_3_^3−^ was intentionally formed through a spontaneous ripening reaction (Equation 11) to regulate the overall oxo‐basicity of the reaction environment.

The overall reaction of initial ripening process:

(11) $$mB_{4}{O_{7}}^{2-}+n{{\mathrm{CO}}_{3}}^{2-}↔x{{\mathrm{BO}}_{3}}^{3-}+y{\mathrm{BO}}_{2}^{-}+z{\mathrm{CO}}_{2}$$

#### 2.1.1 Borate‐Enhanced Cathodic Process and Electrochemical Characterizations

**Figure 2:**
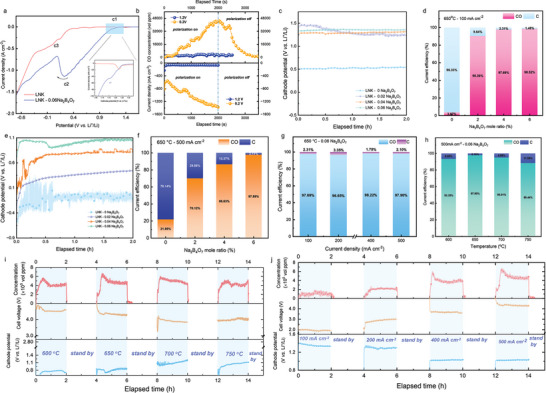
a) Cathodic linear sweep voltammetry (LSV) in different electrolytes. Scan rate: 50 mV s^−1^. b) Potentiostatic electrolysis and product attribution at 1.2 and 0.2 V (vs Li^+^/Li), Galvanostatic electrolysis in LNK with and without borax. The variations of cathodic potential and product distribution at 100 mA cm^−2^ c,d) and 500 mA cm^−2^ e,f). Galvanostatic electrolysis in LNK‐0.06Na_2_B_4_O_7_ at different current densities g,i) and operating temperatures h,j).

To verify our hypothesis, we conducted linear sweep voltammetry (LSV) tests on pure LNK and LNK with borax (LNK‐0.06Na_2_B_4_O_7_). By adjusting initial conditions like atmosphere and temperature, we aimed to control the forms of borate species in the electrolyte. By maintaining a CO_2_ atmosphere, we were able to create a mix of B_4_O_7_^2−^, BO_2_^−^, and BO_3_^3−^ in the electrolyte due to the reversible nature of the ripening process, which is highly sensitive to environmental factors. As can be seen in **Figure** **2**a, unlike LNK which exhibited a single cathodic peak c3 associated with CO_2_ER, LNK‐Na_2_B_4_O_7_ displayed a series of broad cathodic peaks before cathodic peak c3. The onset potential of the cathode process in LNK‐Na_2_B_4_O_7_ was significantly shifted positively by at least 1000 mV compared to LNK, signifying a notable alteration in the thermodynamic reaction pathway due to the addition of Na_2_B_4_O_7_, consistent with our thermodynamic analysis. Two cathodic peaks, c1 and c2, were observed at 1.2 and 0.2 V (vs Li^+^/Li), which are believed to be associated with the involvement of B_4_O_7_^2−^ and BO_2_^−^ in CO_2_ER, respectively. The fluctuating curve suggests the probable formation of gaseous products. To confirm the cathode process and product attribution, potentiostatic electrolysis was respectively conducted at 1.2 and 0.2 V (vs Li^+^/Li). The results (Figure 2b) indicated that CO gas was a prevalent product in LNK‐Na_2_B_4_O_7_, linking cathodic peaks c1 and c2 to CO_2_ER. Conversely, no gaseous product was detected in pure LNK within the potential range of 1.2 to −0.4 V (vs Li^+^/Li). To verify whether adding borax would facilitate CO_2_ER, LSVs under varying borax concentrations (Figure S2a, Supporting Information) were conducted. The results show that the current of the cathodic peak related to CO_2_ER was more evident with increasing borax concentration, which aligned with the findings above.

To investigate the impact of different oxo‐acidic borate species forms on the cathodic process, we manipulated the initial atmosphere (Ar) to favor the ripening reaction Equation (11) and drove the conversion of B_4_O_7_^2−^ to BO_2_^−^. Figure S2b (Supporting Information) displays the cyclic voltammetry (CV) curve of the electrolytes under these conditions. The findings reveal that the presence of oxo‐acidic BO_2_^−^ alone was less advantageous on enhancing cathodic process. Specifically, the cathodic peak associated with CO_2_ER exhibited a more negative onset potential and lower peak current in comparison to the system where B_4_O_7_^2−^and BO_2_^−^ coexisted. This clearly demonstrates that the combination of B_4_O_7_^2−^ with BO_2_^−^ offers greater advantages in reducing cathode polarization than the presence of BO_2_^−^ alone.

Galvanostatic electrolysis was conducted in LNK containing Na_2_B_4_O_7_ to investigate the impact of operating temperature and borax concentration on cathodic product distribution, as well as the maximum current density achievable before reaching the cathodic limit. Figure 2c,d demonstrates the variations of cathodic potential and the distribution of cathodic products at 100 mA cm^−2^. As can be clearly seen, it suffers much more intensive cathodic polarization for borate‐free LNK, reaching 0.5 V (vs Li^+^/Li), where carbon was the primary product. While CO was a dominant product for LNK‐Na_2_B_4_O_7_ electrolytes and the cathodic potentials were just in the vicinity of 1.3 V (vs Li^+^/Li). Furthermore, Figure 2e,f illustrates the performance of electrolytes with varying borax content near the industrial current density of 500 mA cm^−2^ to explore their upper current density limits. Obviously, the original LNK system lacking borax was unable to sustain 500 mA cm^−2^ due to a much more negative cathodic potential −0.2 V (vs Li^+^/Li) than alkali metal deposition potential. By stark contrast, electrolytes with borax exhibit clear advantages. While a low borax concentration (1–5 mol%) cannot support such high current density, increasing borax concentration gradually shifts the cathode potential positively and reduces cathodic polarization. For 6 mol% borax addition (e.g., LNK‐0.06Na_2_B_4_O_7_), the cathodic potential can still be maintained ≈1.0 V (vs Li^+^/Li) at 500 mA cm^−2^, which was considerably 1000 mV before reaching a cathodic limit (e.g., alkali metal deposition). Even at such a high current density, the product selectivity can still be altered. For instance, with a borax content of only 2 mol%, the CO yield exceeded 70% (Figure 2f). When the borax content was raised to 6 mol%, CO became the primary cathode product within the 100–500 mA cm^−2^ current density range (Figure 2g), with a current efficiency exceeding 95%. Notably, the cell voltage was merely 2 V at 100 mA cm^−2^ (Figure 2i; Figure S3a, Supporting Information). Even at a high current density of 500 mA cm^−2^, the CO yield can still reach up to ≈98% (Figure 2g), in good agreement with thermodynamic evaluation. This observation suggests that increasing borax content enhances oxo‐acid borate species, compelling the activity of oxide ions located in the CO‐dominated region (Figure 1b).

Temperature was found to significantly influence the types of borate species present,^[^ ^31^, ^35^ ^]^ with experiments conducted in the 600–750 °C range revealing a current efficiency of over 95% for CO_2_‐to‐CO conversion at 650–700 °C, but less satisfactory results at 750 °C (Figure 2h). This discrepancy was attributed to a “temperature effect,” where higher temperatures led to the conversion of oxo‐acidic borates to oxo‐basic ones, resulting in fewer oxo‐acidic borates available to buffer oxide ions and enhance the process.^[^ ^35^ ^]^ It was concluded that maintaining a relatively high content of acidic borate species is crucial to fully leverage the thermodynamic benefits of borate‐enhanced CO_2_ER, with a recommended optimal temperature of 650 °C for CO_2_‐to‐CO conversion (see much fewer carbon deposited on the cathode in Figure S3c,d, Supporting Information). Overall, the use of oxo‐acidic chemicals promotes the conversion of oxide ions at the cathode, enhancing process kinetics, reducing polarization, and altering product selectivity. Notably, the borate species B_4_O_7_^2−^ exhibited superior conversion performance compared to BO_2_^−^.

### 2.2 Borate‐Enhanced Anodic OER Process

The pathway of the anode reaction not only impacts the overall efficiency of CO_2_ capture and conversion in electrolysis but also influences the energy consumption of the electrolytic process. Taking LNK as an example, the predominant carbonate content in the electrolyte often leads to the anodic reaction being driven by the Oxygen Evolution Reaction (OER) process of carbonate (Equation 12). However, besides producing O_2_, this process also releases additional CO_2_, especially under high polarization conditions such as high current density. This results in a CO_2_/O_2_ ratio close to the theoretical value of 2:1,^[^ ^29^ ^]^ significantly reducing the efficiency of CO_2_ capture and conversion. Moreover, the high electrode polarization required to initiate the OER reaction involving CO_3_^2−^ (theoretical value of 2.89 V vs Li^+^/Li) contributes to increased energy consumption. Ideally, a pure O_2_ generation process originating from oxide ions (Equation 13) at lower polarization conditions (theoretical value of 2.47 V vs Li^+^/Li) is preferred for the anode process. According to the Nernst equation, a higher O^2−^ activity/content necessitates a more negative potential (i.e., less anodic polarization) to activate OER. However, the limited solubility of O^2−^ in traditional electrolytes hinders such attempts, as it can only sustain a limited anode current density that fails to match the high cathodic current density. As anodic polarization increases, the anode reaction gradually shifts toward CO_3_^2−^‐involved OER. Therefore, we here proposed a feasible strategy that chemically transforming O^2−^ into another oxo‐basic group with higher solubility could enhance anodic reaction kinetics at significantly reduced polarization.

CO_3_^2−^‐involved OER:

(12) $$\begin{array}{c} 2C{O_{3}}^{2-}-4e^{-}=2CO_{2}\left(g\right)+O_{2}\left(g\right)\;\;E=2.89\;V\;versus\;Li^{+}/Li \end{array}$$

O^2−^‐involved OER:

(13) $$2O^{2-}-4e^{-}=O_{2}\left(g\right)\;\;E=2.47\;V\;versus\;Li^{+}/Li$$

Borate‐involved OER:

(14) $${\mathrm{mB}}_{x}O_{y}^{z-}-4e^{-}={\mathrm{nB}}_{x^{\prime }}O_{y^{\prime }}^{z^{\prime }-}+O_{2}\left(g\right)$$

(15) $$2{\mathrm{BO}}_{3}^{3-}-4e^{-}=2{\mathrm{BO}}_{2}^{-}+O_{2}\left(g\right)\;\;E^{0}=2.62\;V\;versus\;Li^{+}/Li$$

(16) $$\begin{array}{c} \frac{8}{5}{\mathrm{BO}}_{3}^{3-}-4e^{-}=\frac{2}{5}B_{4}O_{7}^{2-}+O_{2}\left(g\right)\;\;E^{0}=2.70\;V\;versus\;Li^{+}/Li \end{array}$$

(17) $$\begin{array}{c} \frac{3}{4}{\mathrm{BO}}_{3}^{3-}-4e^{-}=\frac{1}{2}B_{3}O_{5}^{-}+O_{2}\left(g\right)\;\;E^{0}=2.69\;V\;versus\;Li^{+}/Li \end{array}$$

(18) $$\begin{array}{c} \frac{10}{7}{\mathrm{BO}}_{3}^{3-}-4e^{-}=\frac{2}{7}B_{5}O_{8}^{-}+O_{2}\left(g\right)\;\;E^{0}=2.72\;V\;versus\;Li^{+}/Li \end{array}$$

**Figure 3:**
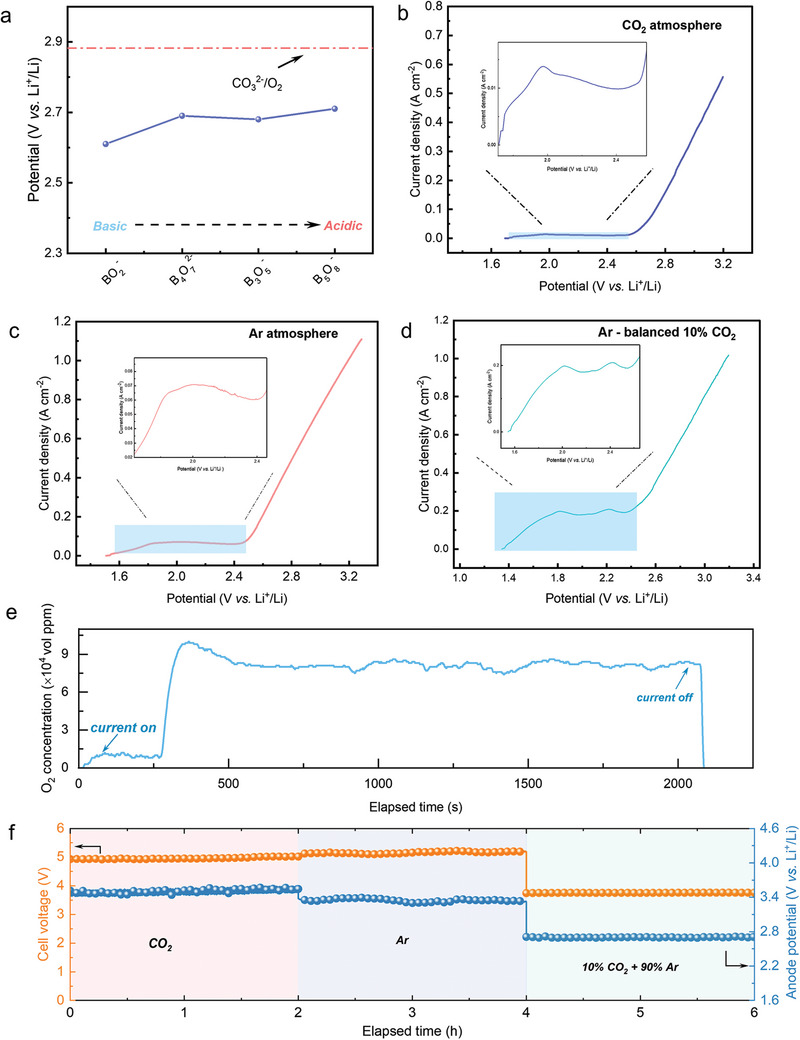
a) Thermodynamic analysis of borate‐involved OER compared to carbonate‐involved OER. Anodic linear sweep voltammetry in LNK‐0.06Na_2_B_4_O_7_ at 650 °C on a FeNi36 anode under CO_2_ b), Ar c), and Ar‐balanced 10%CO_2_ d), respectively. e) The O_2_ content in the anode outlet gas at the anodic polarization of 2.2 V (vs Li^+^/Li). f) The variations of anodic potential and cell voltage during galvanostatic electrolysis at 500 mA cm^−2^ under different atmospheres.

The acidic B_4_O_7_^2−^ and BO_2_^−^ can spontaneously convert the O^2−^ released on the cathode side into the BO_3_^3−^, an oxo‐basic species with solubility at least ten times higher than that of O^2−^.^[^ ^36^ ^]^ Therefore, it is reasonable to conclude that BO_3_^3−^ could potentially serve as an OER precursor to replace O^2−^. The thermodynamic results of borate‐involved OER via Equation (13) and (14) are illustrated in **Figure** **3**a. It is evident that the OER involving BO_3_^3−^ is more negative than that involving CO_3_^2−^, which is beneficial for reducing anode polarization.

To investigate the impact of borate on the anode, LSV tests were conducted on LNK and LNK‐Na_2_B_4_O_7_ in different atmospheres using a preoxidized FeNi36 strip as an inert oxygen evolution anode. The corresponding LSV results are shown in Figure 3b–d. It was observed that the onset potential of LNK‐Na_2_B_4_O_7_ in a 100% CO_2_ atmosphere was 2.5 V (vs Li^+^/Li). However, a negative shift of onset potential was observed when the atmosphere was changed to an Ar atmosphere, this observation was attributed to the presence of oxo‐basic BO_3_^3−^ under the Ar atmosphere, while oxo‐acidic B_4_O_7_^2−^ and BO_2_^−^ are dominant in the CO_2_ atmosphere. Potentiostatic electrolysis was then carried out within the anodic current potential range to confirm the anode reaction pathway. The outlet gas was real‐time monitored during the electrolysis and the results revealed a stable O_2_ concentration for LNK‐Na_2_B_4_O_7_ under the Ar atmosphere (Figure 3e), with no visible corrosion on the anode surface after electrolysis (Figure S4, Supporting Information). These observations indicate that the anode LSV is attributed to OER rather than the corrosion or anodic dissolution of the anode material.

In our previous research, we observed that adjusting the atmosphere over the anode can effectively control the OER pathway of BO_3_^3−^.^[^ ^36^ ^]^ For example, keeping Ar atmosphere leads to the formation of by‐products BO_2_^−^ (Equation 15). Alternatively, when the atmosphere over anode was Ar‐balanced 14% CO_2_, the OER pathway of BO_3_^3−^ results in the formation of by‐products B_3_O_5_^−^ and B_5_O_8_^−^ (Equations 17 and 18). These observations suggest that the as‐formed borate species after OER can be manipulated by precisely regulating the microenvironment at the anode. In this work, to establish a self‐looping process involving B_4_O_7_^2−^, BO_2_^−^, and BO_3_^3−^, we aimed to adjust the anode atmosphere to facilitate the anodic OER process where BO_3_^3−^ is converted to B_4_O_7_^2−^ (Equation 16). This conversion pathway is expected to compensate for the B_4_O_7_^2−^ that are consumed at the cathode, keeping the mass balance and making the electrolysis more sustainable. Given consideration that the oxo‐acidity of B_4_O_7_^2−^ falls between that of BO_2_^−^ (Ar atmosphere preferentially formed) and B_5_O_8_^−^ (Ar‐balanced 14%CO_2_ atmosphere, it is reasonable to infer that an OER pathway occurs under an intermediate atmosphere (e.g., Ar‐balanced 10% CO_2_) would yield B_4_O_7_^2−^. Figure 3f demonstrate the anodic behavior in LNK‐Na_2_B_4_O_7_ under Ar‐balanced 10% CO_2_, with a significantly improved anodic current plateau. The potentiostatic electrolysis conducted within this potential range indicated that the anodic process remains an oxygen evolution process.

In order to verify the OER enhancement effect of borate on anode under industrial conditions, the anodic current density of 500 mAcm^−2^ which is identical to the cathodic one was applied. In addition, to explore the influence of the transformation rule of borate species at the anode side during electrolysis, galvanostatic electrolysis was carried out sequentially under CO_2_, Ar, and Ar‐balanced 10% CO_2_ atmospheres, where the variations of anodic potential and cell voltage under different atmospheres were real‐time monitored (Figure 3f). It should be noted that we isolated the cathode with an alumina tube to control the atmospheres of the cathode chamber and the anode chamber individually. As shown in Figure 3f, the results show that the anode polarization potential was the most positive when the anode atmosphere was CO_2_, which may be because the borates in the electrolyte were mostly acidic borates (B_4_O_7_^2−^, BO_2_^−^) in the CO_2_ atmosphere, and the content of oxo‐basic borates that can trigger the anode enhancement effect was relatively low. When the anode atmosphere sequentially shifted to the Ar atmosphere, the anodic potential shifted negatively by ≈100 mV due to the increased proportion of oxo‐basic borates in the electrolyte. In this case, however, it is worth noting that even if the polarization of the anode is reduced, the overall cell voltage was increased by ≈0.2 V, which was probably because the OER pathway of BO_3_^3−^ was to generate BO_2_^−^. In other words, at this time, the acidic borate in the electrolyte is mainly BO_2_^−^, and the effect of its cathode enhancement effect was weaker than that of B_4_O_7_^2−^. Due to the difference in the contribution of each borate species (oxo‐acidic and oxo‐basic natures) to the anode and cathode, the cell voltage increases.Interestingly, switching the atmosphere to Ar‐balanced 10% CO_2_ resulted in a negative shift of ≈0.65 V in anode polarization, while also reducing the cell voltage by 1.28 V compared to Ar, reaching 3.85 V. This shift is attributed to the presence of oxo‐basic BO_3_^3−^ in the anode atmosphere, which enhances the anode process and facilitates the OER pathway of BO_3_^3−^, leading to the generation of B_4_O_7_^2−^. The migration of B_4_O_7_^2−^ from the anode side to the cathode side helps reduce cathode polarization. These results align well with electrochemical measurements, demonstrating the significant influence of borate species on both anode and cathode processes for CO_2_ electrolysis. By controlling the atmospheres of the anode and cathode separately, a balanced distribution of B_4_O_7_^2−^, BO_2_^−^, and BO_3_^3−^ in the electrolyte can be achieved, promoting efficient cathode and anode processes through the distinct functions of borates and resulting in a substantial reduction in cell voltage. Based on the findings above, we additionally conducted the content variation of anodic oxygen evolution during CO_2_ electrolysis under varying current densities, meanwhile the CO_2_ concentration at the anode was also monitored. As shown in Figure S5 (Supporting Information), the oxygen concentration was increased with increasing current density, while no evident changes (background content only) of CO_2_ concentration was observed at the anode, suggesting a net CO_2_ conversion efficiency of ≈100%.

### 2.3 Borate‐Enhanced CO_2_ Electrolysis and Long‐Term Stability

**Figure 4:**
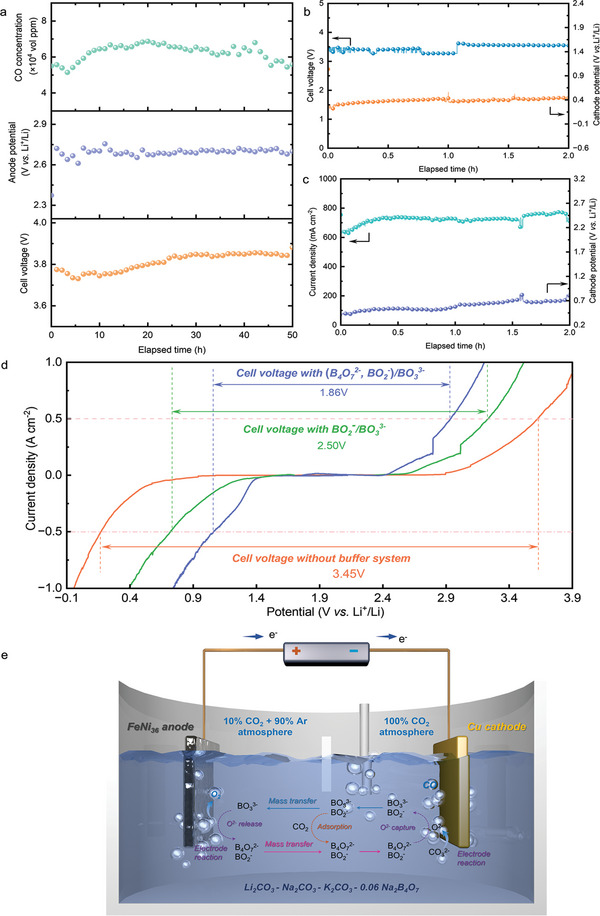
Galvanostatic electrolysis at 500 mA cm^−2^ in LNK‐0.06Na_2_B_4_O_7_ at 650 °C on a Cu plate cathode a) and a Cu foam cathode b), respectively. c) Constant‐voltage electrolysis in LNK‐0.06Na_2_B_4_O_7_ at 650 °C on a Cu foam cathode. d) The calibrated cell voltage based on LSV under optimal conditions. Scan rate: 1 mV s^−1^. e) The mechanism overview of borate‐enhanced molten salt CO_2_ electrolysis.

To gain more insights, we further explored the conditions and influencing factors to concurrently realize the enhancement effect on the cathode side and the anode side. In order to verify the long‐term stability of the borate buffer system to enhance the molten salt CO_2_ capture and electrolysis technology, we carried out galvanostatic electrolysis in LNK‐0.06Na_2_B_4_O_7_ electrolyte at 500 mA cm^−2^ current density for 50 h, where the variations of cell voltages, corresponding anode and cathode potentials were recorded in real time. A common Cu plate was used for the cathode, and the atmosphere of the cathode chamber was pure CO_2_, while the anode was a pre‐oxidized FeNi36 strip, and the atmosphere of the anode chamber was Ar‐balanced 10% CO_2_. As shown in Figure 4a, during the 50 h electrolysis process, the cell voltage and anode potential can still be stable at 1.85 V and 2.6 V (vs Li^+^/Li), respectively, indicating that the mass balance of borate species participating in CO_2_ER and OER can be ensured, establishing a stable borate‐mediated looping (Figure 4e). In addition, the cathodic product was CO, with a current efficiency of more than 98%. It should be pointed out that a non‐nanostructured Cu plate cathode was initially used in the experiment. Furthermore, a series of experiments were concuted to evaluate the morphological and compositional changes of the electrolysis system after the 50‐hour electrolysis. To be specific, we conducted LSV before and after long‐term CO_2_ electrolysis (Figure S4a, and the results show that the LSVs were almost overlapped, indicating that the electrolyte remained stable after 50 h electrolysis, this observation was in good agreement with ICP result (see negligible metal content variation in the electrolyte before and after electrolysis) (Table S1). After CO_2_ electrolysis for 50 h, we found that the anode was still dimensionally stable (Figure S4b, c). In addition, we analyzed the morphological changes of the anode by ultra‐depth field microscopy (VHX‐5000, China) (Figure. S4d–e), we also conducted XRD analysis on the electrodes (Figure. S4f, g). The above‐mentioned results clearly show neither evident morphological nor compositional changes were observed after 50 h electrolysis.

To further maximize the benefits of the borate buffer system, the original Cu plate was replaced with a Cu foam of equivalent geometric area. Electrolysis was conducted at a constant pressure of 500 mA cm^−2^ and 3.85 V, which was consistent with the conditions used for the Cu sheet. For galvanostatic electrolysis at 500 mA cm^−2^, the cathode potential was 1.2 V (vs Li^+^/Li) and the cell voltage was 3.5 V (Figure 4b). Interestingly, during constant‐voltage electrolysis at 3.85 V (Figure 4c), the current density could exceed 700 mA cm^−2^ with the cathodic potential at ≈0.7 V (vs Li^+^/Li), indicating that altering the electrode structure could further enhance the upper limit of cathodic reaction kinetics. The use of an alumina tube to separate the cathode from the anode led to an inevitable increase in the IR drop of the electrolytic system.

To demonstrate the intrinsic thermodynamic advantages of borate‐enhanced CO_2_ electrolysis, the applied cell voltage was further calibrated by a slow‐scanning LSV test without using alumina tube (Figure S1a, Supporting Information) under the repective optimal atmosphere. The results revealed that even at a current density of 500 mA cm^−2^, the cell voltage was merely 1.86 V (Figure 4d), much lower than that of borate‐free LNK and oxo‐acidic BO_2_^−^‐alone systems. By respectively regulating the atmosphere to tailor the borate species (B_4_O_7_^2−^, BO_2_^−^, and BO_3_^3−^ co‐existed), both the CO_2_ER and OER processes were kinetically enhanced. Even at an industrial current density of 500 mA cm^−2^, the CO selectivity reached 98%, meanwhile, the energy efficiency remained as high as 55.32%.

### 2.4 Comparison with Other CO_2_ER Systems

**Figure 5:**
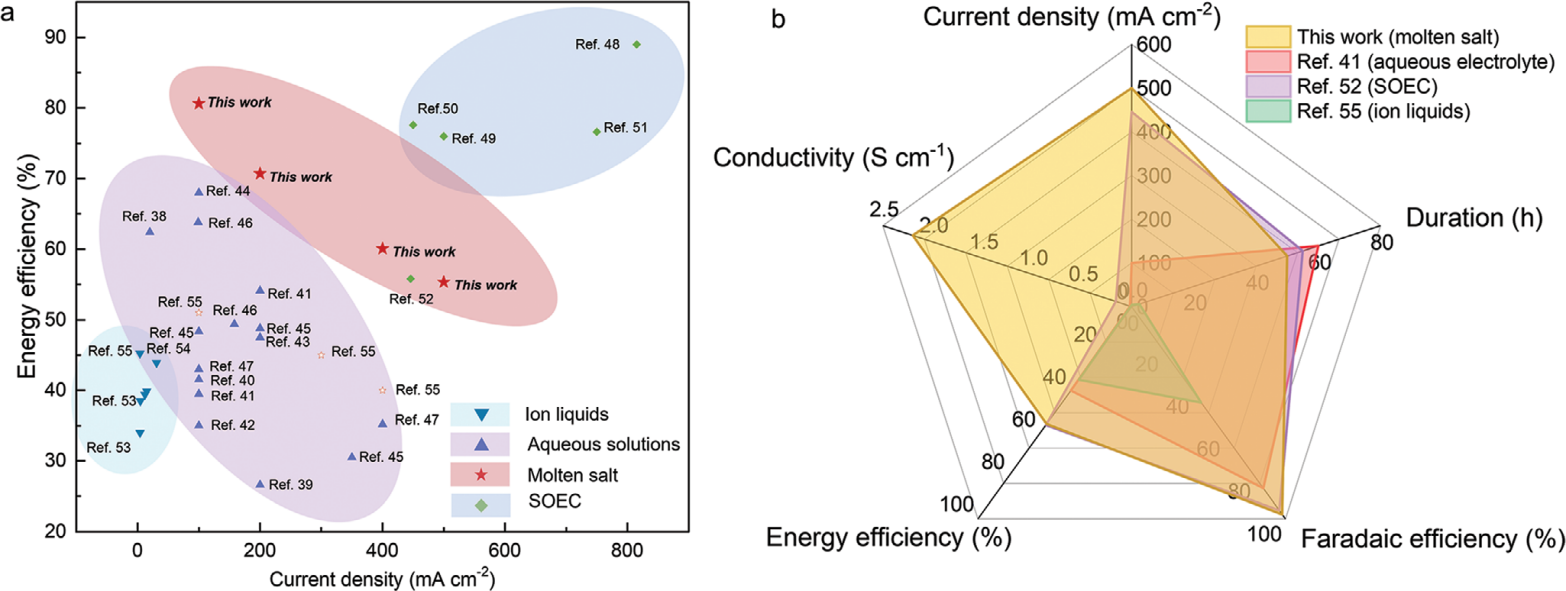
Overview comparison of typical CO_2_ER systems a) Energy efficiency perspective; b) Comparison of the performance factor using different electrolytes.

We classified our findings based on various electrolyte systems and compared the progress of CO₂ conversion to CO across several dimensions: current density, Faradaic efficiency, energy efficiency, duration, and conductivity, the overview comparisons are illustrated in **Figure** **5** and the detailed information are listed in Table S2 (Supporting Information).^[^ ^38^, ^39^, ^40^, ^41^, ^42^, ^43^, ^44^, ^45^, ^46^, ^47^, ^48^, ^49^, ^50^, ^51^, ^52^, ^53^, ^54^, ^55^ ^]^

As indicated in Figure 5 and Table S2 (Supporting Information), compared to aqueous electrolytes, our approach significantly outperforms in terms of current density and energy efficiency, without relying on costly and complex electrocatalysts. The potentials observed at a universal current density exhibit markedly lower polarizations compared to those within a similar current range in aqueous solutions. This finding suggests that the energy consumption for borate‐enhanced molten salt CO_2_ electrolysis is advantageous. To highlight our exceptional energy‐transforming performance, we normalized the potential (for both CO_2_ER and OER) to the Normal Hydrogen Electrode (NHE), facilitating easier comparisons among different electrolysis systems and clearly illustrating the advantages of our work (Table S3, Supporting Information). As a result, the presence of borate‐containing electrolyte significantly enhances the performance of CO_2_ electroreduction (CO_2_ER) and oxygen evolution reaction (OER). For instance, at a current density of 100 mA cm^−^^2^, the cathodic potential reached only −0.748 V (vs NHE), in contrast to −1.291 V (vs NHE) for the aqueous solution. Furthermore, at a current density of 500 mA cm^−^^2^, the cathodic polarization was 381 mV lower than one of the most advanced results reported in aqueous solutions (452 mA cm^−^^2^, −1.410 V vs NHE).^[^ ^56^ ^]^

In contrast to the solid oxide electrolysis cell (SOEC), our method does not depend on rare elements such as Y, Zr, La, Sr, and Ga, and operates within a lower temperature range. Furthermore, when compared to ionic liquids, we demonstrate clear advantages in current density, Faradaic efficiency, and energy efficiency. Considering that cost‐effectiveness is also critical to CO_2_ER technology, we also evaluated the cost implications among typical CO_2_ electrolysis systems (Table S4, Supporting Information). Our findings reveal that the cost of molten salt CO_2_ electrolysis for CO production in this study demonstrates significant economic advantages, attributed to the use of inexpensive electrolytes and cost‐effective electrodes.

## 3 Conclusion

In this paper, we developed a borate‐enhanced molten salt carbon dioxide capture and electrochemical conversion process to achieve CO_2_‐to‐CO conversion on a non‐nanostructured transition metal plate electrode at 650 °C with a high selectivity of over 98%, where CO_2_ER was coupled with OER at an overall current density of 500 mA cm^−2^, reaching over 55% energy efficiency without the need for complex and expensive electrocatalysts. By adding cheap and earth‐abundant borax (Na_2_B_4_O_7_) into the conventional carbonate electrolyte to adjust the oxo‐basicity of the electrolyte, we naturally create a buffered electrolyte that can drive the electrode reaction faster and more efficiently. The entire electrochemical reaction, by manually regulating the comprehensive basicity of the reaction environment, induces the generation of target borates (BO_3_^3−^, BO_2_^−^, and B_4_O_7_^2−^) with their specific functions. These borates can constantly shuttle between the cathode and the anode, maintaining their relative concentration, where the oxo‐acidic borate species (BO_2_^−^ and B_4_O_7_^2−^) can combine with the released oxide ions on the cathode side, this electrode interface process drives CO as the main cathode product. The more oxo‐basic borate species (BO_3_^3−^) not only can capture CO_2_ in the atmosphere but also serve as a precursor to participating in the anodic OER, thereby further kinetically improving OER performance. In addition, controlling the atmosphere over the anode can force spontaneous directional transformation of the borate, keeping the composition and form of borates in the electrolyte relatively stable with long‐term operating stability of 50 h accumulatively. This work proposes an economically feasible strategy: by establishing a sustainable borate‐mediated closed loop, CO_2_ can be directionally converted to CO at an industrial scale with a reduced carbon footprint.

## 4 Experimental Section

### Materials and Electrolyzer Configuration

A mixture of anhydrous Li_2_CO_3_, Na_2_CO_3_, and K_2_CO_3_ (Sinopharm Chemical Reagent Co. Ltd, purity >99%) was chosen as a supporting electrolyte, in which Na_2_B_4_O_7_ (Shanghai Maclean's Biochemical Technology Co., Ltd, purity >99.9%) as an additive was introduced into the electrolyte to change the pristine thermodynamic pathways of electrochemical reactions. To be specific, a corundum (γ‐Al_2_O_3_) crucible containing 500 g of Li_2_CO_3_‐Na_2_CO_3_‐K_2_CO_3_ (43.5: 31.5: 25 in molar ratio, a melting point of 397 °C) with/without 6 mol% (if elsewhere specified) Na_2_B_4_O_7_ was put into a sealed vertical tube furnace. Afterward, the salt mixture was dried under an Ar atmosphere at 300 °C for 24 h to remove the adhered moisture. Then, it was heated up to the target operating temperature (600–750 °C) under a pure CO_2_ atmosphere. To verify the enhancing effect of borate additives toward both CO_2_RR and OER, Li_2_CO_3_‐Na_2_CO_3_‐K_2_CO_3_ carbonate mixtures (denoted as LNK) with a series of Na_2_B_4_O_7_ contents (from 0 to 6 mol% based on the molar ratio (*x*) between Na_2_B_4_O_7_ and LNK) were prepared for the experiments, named as LNK–*x*Na_2_B_4_O_7_ (e.g., LNK–0.06Na_2_B_4_O_7_). To highlight the genuine current density associated with the improvement of electrolyte composition, a smooth Cu plain sheet (1.5 cm × 1.5 cm, purity >99.9%) was used as the cathode, while a smooth FeNi36 plain sheet with equal dimensional size (1.5 cm × 1.5 cm, purity >99.0%) was used as the anode (Figure S1a, Supporting Information). To facilitate the collection of cathodic and anodic products and to regulate the atmosphere over both the cathode and anode, an open‐ended cylindrical alumina tube (Φ 33 cm) was employed to encase the cathode. This design effectively separates the cathode chamber from the anode chamber while still allowing for mass transfer of ions (Figure S1b, Supporting Information). Due to the high sensitivity of the borate‐containing electrolyte to atmospheric conditions,^[^ ^31^ ^]^ CO_2_ was bubbled into the electrolyte for a minimum of 2 h prior to each experiment to restore the initial composition, unless otherwise specified.

### Electrochemical Measurements

Prior to electrolysis, electrochemical measurements were carried out on an electrochemical workstation (CS310, Wuhan Corrtest Instruments Co., China) using a three‐electrode system. To elucidate the mechanism of electrode reaction, cyclic voltammetry (CV) and linear sweep voltammetry (LSV) were respectively used. In detail, for CO_2_ER, a smooth Cu wire (1 mm in diameter, contact area 0.32 cm^2^) and a smooth FeNi36 sheet (3 cm × 3 cm, contact area 9 cm^2^) served as a working electrode (WE) and a counter electrode (CE), respectively. For OER, a FeNi36 strip (1 mm in diameter, contact area 0.32 cm^2^) acted as WE, while a Cu sheet (3 cm × 3 cm, contact area 9 cm^2^) acted as CE. The reference electrode (RE) was a silver wire inserted in a mullite tube (Pythagoras type), which was pre‐filled with 0.5 mol% Ag_2_SO_4_‐containing LNK. The electrochemical experiments were carried out under different atmospheres with various contents of Na_2_B_4_O_7_, where the atmosphere was kept over the melt by a flow (200 mL min^−1^) of the specified gas (e.g., Ar, Ar‐balanced 10 vol% CO_2_, and pure CO_2_) using a mass flow controller (MF‐4701‐RF, Beijing Sevenstar flow Co. Ltd, China) in either cathode chamber or anode chamber. To verify the product attribution, chronoamperometry was carried out and the corresponding electrolysis products were collected and analyzed.

### CO_2_ Electrolysis

Galvanostatic electrolysis was performed in LNK with and without Na_2_B_4_O_7_ for 2 h at 650 °C using a computer‐controlled DC power source (Shenzhen Neware Electronic Ltd., China). To reveal the genuine current density associated with the improvement of electrolyte composition, a smooth Cu plain sheet (1.5 cm × 1.5 cm, purity >99.9%, contact area 4.5 cm^2^) was used as the cathode, while a smooth FeNi36 plain sheet with equal dimensional size (1.5 cm × 1.5 cm, purity >99.0%, contact area 4.5 cm^2^) was used as the anode. During the CO_2_ electrolysis, the gaseous products either from the cathode or from the anode were in real‐time monitored.

To gain more insights into the long‐term stability of the borate‐enhanced electrolysis system, galvanostatic electrolysis 500 mA cm^−2^ was carried out at 650 °C in LNK–0.06Na_2_B_4_O_7_ for 50 h, during which the variations of applied cell voltage, electrode polarization, and CO concentration were recorded. To further highlight the promotion of reaction kinetics, a porous Cu foam (1.5 cm × 1.5 cm, purity >99.9%, geometric area 4.5 cm^2^), as a substitute for the smooth Cu sheet, was used to achieve a higher current density at the same applied cell voltage as that using the smooth Cu sheet cathode at 500 mA cm^−2^.

### Current Efficiency and Product Characterizations

The CO concentration and flow rate of the outlet gas were real‐time detected by a gas analyzer (THA100s, Beijing Taihechuanglian Technology Co., Ltd., China) and a mass flow under room temperature (≈25 °C), respectively. The produced CO yield (ξ, mol) and cathodic current efficiency (η_CO_, %) are calculated as follows:

(19) $$\xi_{\mathrm{CO}}=\frac{\int_{0}^{t}cVdt}{V*}$$

(20) $$\eta_{\mathrm{CO}}=\frac{\mathrm{nF}\xi_{\mathrm{CO}}}{Q}\;\times 100$$

where *n* refers to the electron transfer number to produce CO from CO_2_ (*n *= 2), *F* denotes Faraday's constant (9.6485 × 10^4^ C mol^−1^), *c* (ppm), and *V* (mL s^−1^) stands for the recorded CO concentration and outlet flow rate at any time *t*; *V* ^*^ (mL mol^−1^) represents the molar volume at room temperature, *Q* (C) is the overall charge quantity during the electrolysis.

## Conflict of Interest

The authors declare no conflict of interest.

## Supporting information



Supporting Information

## Data Availability

The data that support the findings of this study are available from the corresponding author upon reasonable request.
