# Supplementary material for: Tailoring Borate Mediator Species Enables Industrial CO Production with Improved Overall Energy Efficiency by Sustainable Molten Salt CO2 Electrolysis
Source: Adv Sci (Weinh). 2024 Dec 4;12(4):2406457. doi: 10.1002/advs.202406457 (PMC11775544; doi:10.1002/advs.202406457)
Supplement: Supplementary file 1 — Supporting Information [file ADVS-12-2406457-s001.docx]

**Supporting Information**

**Tailoring Borate Mediator Species Enables Industrial CO Production with Improved Overall Energy Efficiency by Sustainable Molten Salt CO_2_ Electrolysis**

Xinyu Li^1,2^, Bowen Deng^1,2*^, Kaifa Du^1,2^, Wenmiao Li^1,2^, Di Chen^1,2^, Xin Qu^1,2^, Fangzhao Pang^1,2^  Xiaodan Zhang^1,2^, Hao Zha^1,2^, Huayi Yin^1,2^, Dihua Wang^1,2,3*^

1. School of Resource and Environmental Science, Wuhan University, 299 Bayi Road, Wuchang District, Wuhan 430072, P. R. China.
2. Hubei International Scientific and Technological Cooperation Base of Sustainable Resource and Energy, Wuhan University, Wuhan 430072, China
3. State Key Laboratory of Water Resources and Hydropower Engineering Science, Wuhan University, Wuhan 430072, China

Email: bwdeng@whu.edu.cn; wangdh@whu.edu.cn

**Experimental**

**Thermodynamic analysis**

The thermodynamic equilibrium potential (the activity of each component in the equation is considered to be 1) of the reaction can be calculated as following: (**Eq. S1**):

$$\begin{aligned} E^{0}=\frac{\Delta_{r}G^{⊖}}{nF}\#\left( S1 \right) \end{aligned}$$

$E^{0}$is the thermodynamic equilibrium potential, V; $\Delta_{r}G^{⊖}$is the Gibbs free energy change, J/mol ; $n$ is the number of electrons transferred; F is the Faraday’s constant, 9.6485×10^4^ C mol^-1^.The Gibbs free energy changes of the reactions are calculated or estimated by software HSC 9.0 (Metso, Finland).

**Gibbs free energy balance**

CO_2_ absorption:

$5CO_{2}+4BO_{3}^{3-}=5CO_{3}^{2-}+B_{4}O_{7}^{2-}$ $\Delta_{r}G_{a}= -131.7kJ mol^{-1}$ (S2)

Cathodic reaction:

$5CO_{3}^{2-}+{2B}_{4}O_{7}^{2-}+10e^{-}=5CO+8BO_{3}^{3-} \Delta_{r}G_{C}=29.0 kJ mol^{-1}$ (S3)

Anodic reaction:

$8BO_{3}^{3-}-20e^{-}=2B_{4}O_{7}^{2-}+5O_{2} \Delta_{r}G_{A}=2228.225kJ mol^{-1}$ (S4)

Overall reaction:

${2CO}_{2}=2CO+O_{2} \Delta G=404.5kJ mol^{-1}$ (S5)

The Gibbs free energy (∆_r_G) of each reaction was calculated based on corresponding Gibbs free energy of formation (∆_f_G) by following equation:

$\Delta_{r}G= \sum\nu\Delta_{f}G$ (S6)

The Gibbs free energy of the overall reaction ($\Delta_{r}G_{\mathrm{overall}}$) is calculated according to the following equation:

$\Delta_{r}G_{\mathrm{overall}}=\frac{2}{5}\Delta_{r}G_{a}+\frac{2}{5}\Delta_{r}G_{C}+\frac{1}{5}\Delta_{r}G_{A}$ (S7)

where $\Delta_{r}G_{a}$, $\Delta_{r}G_{C}$ and $\Delta_{r}G_{A}$represents the Gibbs free energy of CO_2_ absorption, cathodic reaction and anodic reaction, respectively.

**Theoretical potential *vs.* activity of oxide ions**

The correlations between standard theoretical CO_2_ER potentials (*E*) of different products and O^2-^ activity ($a_{O^{2-}}$) were evaluated by following Nernst equations, respectively:

For CO_2_-to-C conversion: CO_3_^2-^ + 4e^-^ = C + 3O^2-^

$$\begin{aligned} E=E^{0}-\frac{RT}{nF}\ln\left( \frac{\left( a_{O^{2-}} \right)^{3}}{a_{{CO}_{3}^{2-}}} \right)=E^{0}-\frac{3RT}{nF}\ln a\left( O^{2-} \right)=E_{0}-\frac{3\cdot2.3RT}{nF}\lg a_{O^{2-}}\#\left( S8 \right) \end{aligned}$$

For CO_2_-to-CO conversion: CO_3_^2-^ + 2e^-^ = CO (g) + 2O^2-^

$\begin{aligned} E=E^{0}-\frac{RT}{nF}\ln\left( \frac{\left( \frac{p\left( CO \right)}{p^{0}} \right)\cdot\left( a_{O^{2-}} \right)^{2}}{a_{{CO}_{3}^{2-}}} \right)=E^{0}-\frac{2RT}{nF}\ln a_{O^{2-}}=E_{0}-\frac{2\cdot2.3RT}{nF}\lg a_{O^{2-}}\#\left( S9 \right) \end{aligned}$

where E represents for Nernst potential, $a$refer to activities of corresponding ions.$P_{CO}$ and $P_{atm}$ denote to CO partial pressure and standard ambient pressure (i.e., $P_{atm}=1 bar$), respectively. *n* stands for electron transfer number, *F* refers to Faraday constant (96480 C/mol), and *R* represents gas constant (8.314 J K^−1^ mol^−1^). To simplify the calculation, $a_{{CO}_{3}^{2-}}$ and $P_{CO}$ are deliberately assumed to be under standard state (i.e., $a_{{CO}_{3}^{2-}}=1$, $P_{CO}=1 bar$), which can reveal the intrinsic thermodynamic tendency related to $a_{O^{2-}}$ only.

**Energy efficiency**

The energy efficiency ($\text{ε}$) is defined by the following equation:.

$\text{ε=}\frac{Stored Energy}{Supplied Actual Energy}\text{=}\frac{U^{0}}{U}\cdot\eta$ (S10)

$U^{0}$(V) is the theoretical voltage (1.05 V at 650 ^o^C) to drive the reaction, and U (V) refers to the practical applied cell voltage. $\eta$ stands for current efficiency toward CO product. Considering the self-generated Joule heat is sufficient to compensate for heat loss and maintain the electrolyte temperature during electrolysis^[1,2]^, particularly at industrial current density level, the initial energy input to raise the temperature of the electrolyte is neglected.

**Potential normalization**

The electrode potentials were directly measured by Ag/Ag_2_SO_4_ reference electrode, which were further presented against Li^+^/Li based on onset potential (*vs.* Ag/Ag_2_SO_4_) of anodic limit (CO_3_^2-^/CO_2_-O_2_) plotted from LSVs (Fig. 4d). To be specific, the potential of Li^+^/Li is 2.89 V negative than that of CO_3_^2-^/CO_2_-O_2_ according to Eq. S1.

CO_3_^2-^ = CO_2_ (g) + O_2_(g) + 4e^-^ E^0^ = 2.89 V vs Li^+^/Li

To safely compare the obtained results (e.g., potential, overpotential) with that in aqueous solutions, the potential (*vs.* Li^+^/Li) was further normalized to normal hydrogen electrode (*vs.* NHE) at room temperature (25 ^o^C) by following equation:

2Li + 2H_2_O = 2LiOH + H_2_ ∆_r_G = -403.554 kJ mol^-1^

2Li = 2Li^+^ + 2e^-^ E^0^ = -2.09 V (vs. H_2_O/H_2_)

2H_2_O + 2e^-^ = H_2_ (g) + 2OH^-^ E^0^ = -0.83 V (vs. NHE)


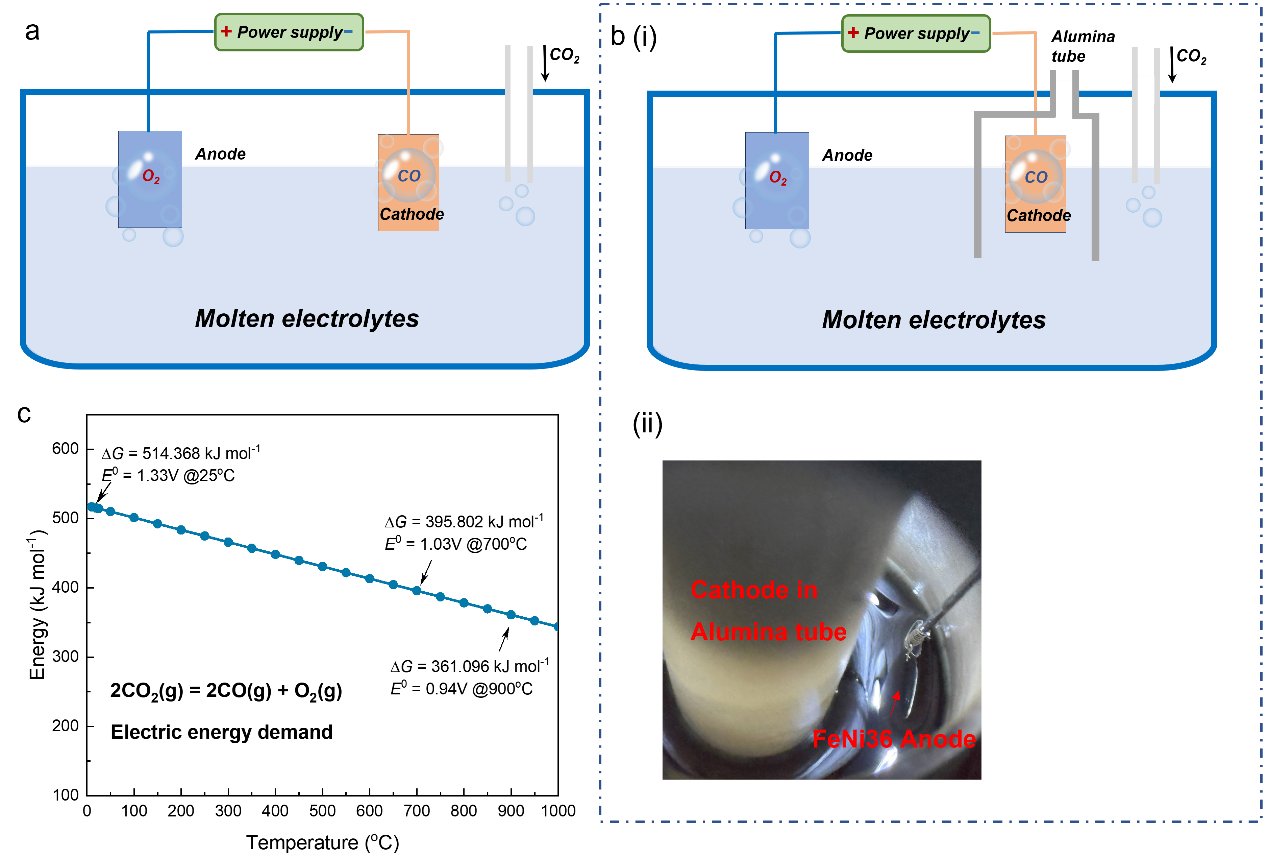


**Figure S1.** Schematic of molten salt electrolyzer configuration with (a) and without (b) alumina tube separating cathode chamber. (c) Gibbs energy change of electrochemical CO_2_-to-CO conversion at varying temperatures.


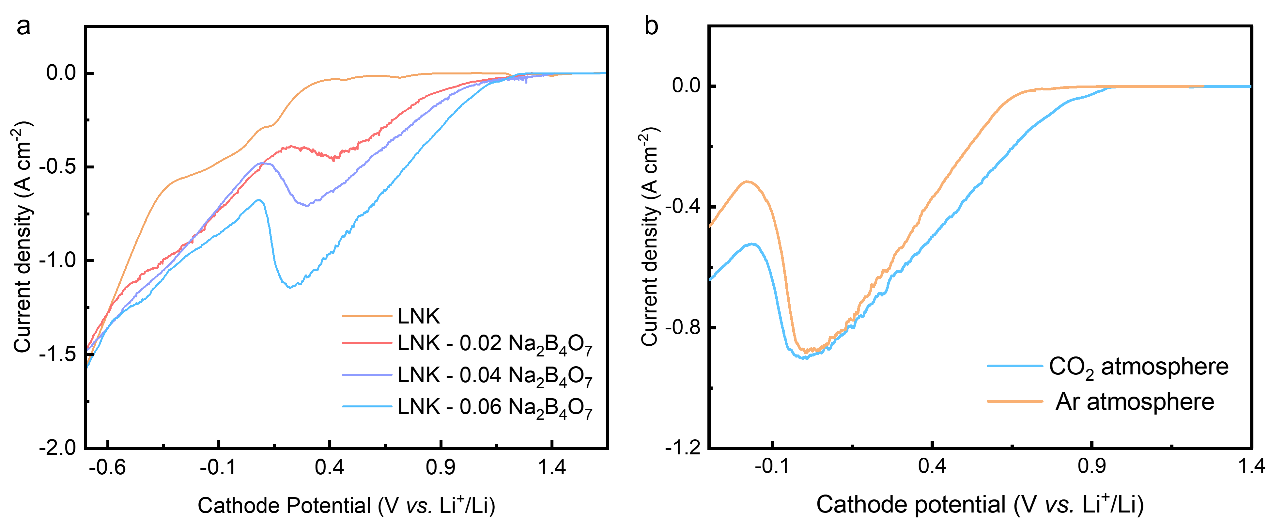


**Figure S2**. (a) Linear sweep voltammetry in LNK with varying contents of Na_2_B_4_O_7_ at 650 ^o^C. Scan rate: 50 mV (b) Linear sweep voltammetry in LNK-0.04 Na_2_B_4_O_7_ at 650 ^o^C under different atmospheres. Scan rate: 50 mV s^-1^.


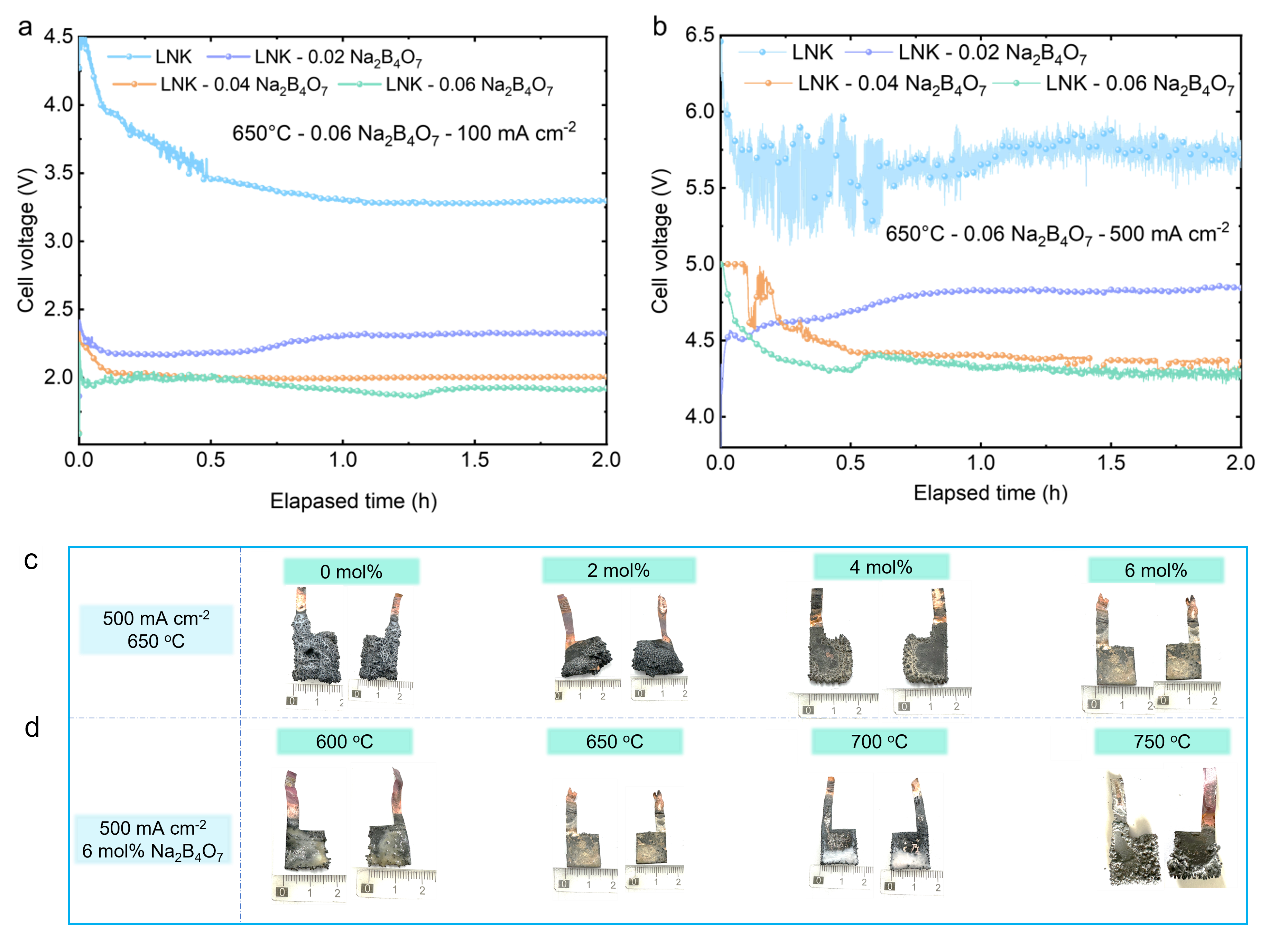


**Figure S3.** The real-time cell voltage in LNK containing varying concentration of Na_2_B_4_O­_7_ during galvanostatic electrolysis at 650 ^o^C. (a) 100 mA cm^-2^. (b) 500 mA cm^-2^.The optical images of the cathode after CO_2_ electrolysis at 500 mA cm^-2^ under varying Na_2_B_4_O_7_ contents (c) and temperatures (d).

**
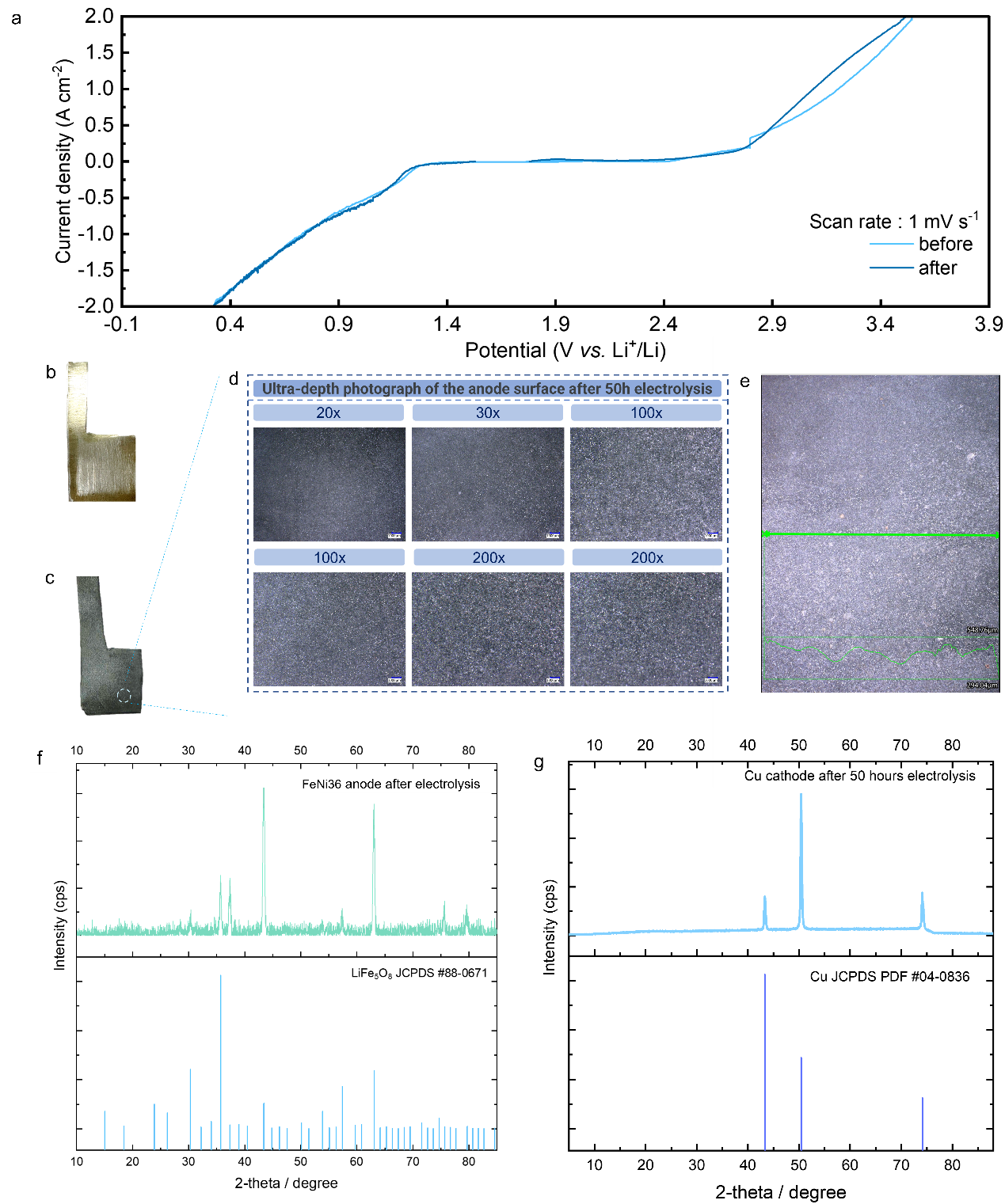
**

**Figure S4.** Stability of the molten salt CO_2_ electrolysis system. (a) Liner sweep voltammetry in molten LNK–0.06 Na_2_B_4_O_7_ before and after long-term CO_2_ electrolysis. Optical images of FeNi36 anode before (b) and after (c) electrolysis. (d-e) Morphology of the anode after long-term CO_2_ electrolysis by ultra-depth field microscopy. XRD patterns of the anode (f) and the cathode (g) after the long-term CO_2_ electrolysis.


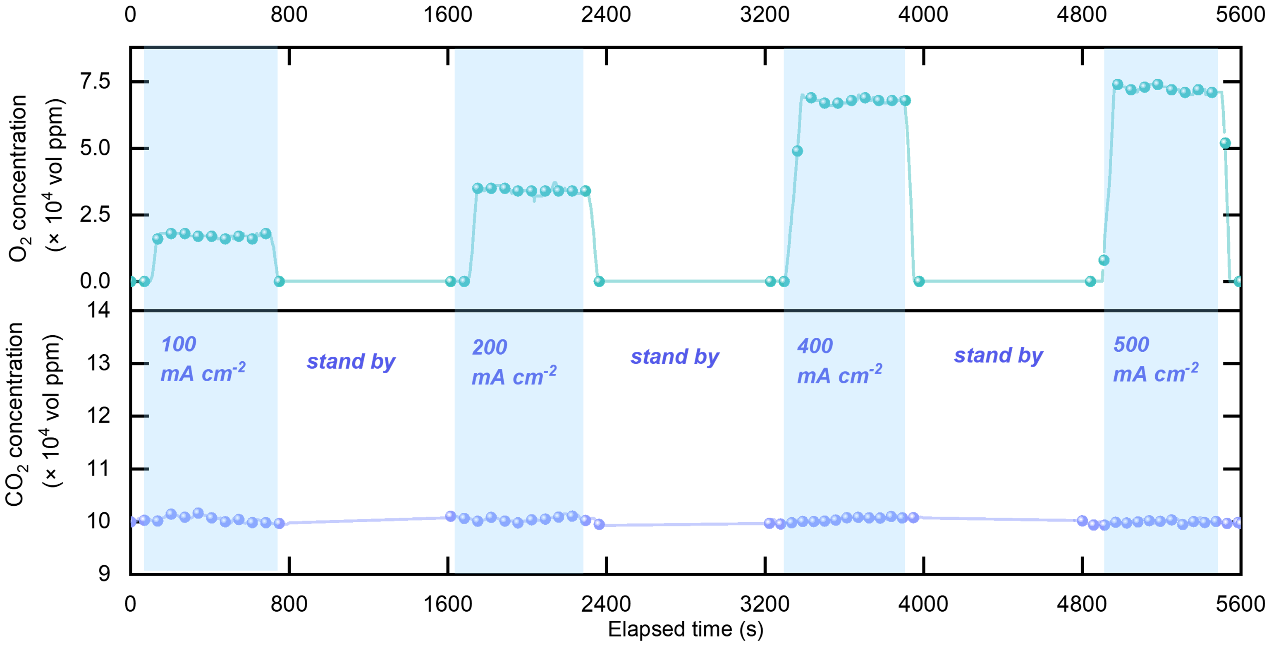
**Figure S5**. The content variations of O_2_ and CO_2_ at the anode.

**Table S1**. Concentrations of Fe and Ni in the electrolyte after 50 h electrolysis

| Element | Pristine (ppm) | After electrolysis  (ppm) |
| --- | --- | --- |
| Fe | 0.8049 | 1.5906 |
| Ni | 10.9601 | 19.5163 |

**Table S2**. Overview comparisons of CO_2_ electroreduction in different electrolytes

| **Electrolytes Types** | **Electrolytes** | **Electrocatalyst** | **Current density (mA cm^-2^)** | **Duration(h)** | **Faradic Efficiency (%)** | **Energy Efficiency (%)** | **Temperature(℃)** | **References** |
| --- | --- | --- | --- | --- | --- | --- | --- | --- |
| Molten salt | LNK-0.06Na_2_B_4_O_7_ | Cu plate | 500 | 50 | 97.9 | 55.32 | 650 | **This work** |
|  |  |  | 100 | - | 97.69 | 81.86 |  |  |
|  |  |  | 200 | - | 96.65 | 70.70 |  |  |
|  |  |  | 400 | - | 98.22 | 60.23 |  |  |
|  | Li_2_CO_3_ | Ti | 100 | - | ~100 | - | 900 | [3] |
|  | CaCO_3_-CaCl_2_ | Stainless steel pipe | 150 | 5.55 | 36.9 | 11.14 | 900 | [4] |
| **Aqueous Solutions** | Cathode: 0.5 M KHCO_3_  Anode: 1 M KOH | antimony-copper single-atom alloy catalyst (Sb1Cu) | 452 (partial) | - | 90.4 | - |  | [5] |
|  | CO_2_-saturated 0.5 M KHCO_3_ | Fe−N_4_/Fe−N_2_ interface on the NP surface | ~20 | 100 | ~90 | 62.4 |  | [6] |
|  | 1 M Cs_2_SO_4_ | Gold/GDL | 200 | 1 | 80-90 | 26.6 |  | [7] |
|  | 0.5 M allyl alcohol + 0.05 M KOH anolyte | Ag/GDL | 100 | 10 | (96 ± 1) | 41.56 |  | [8] |
|  | 1 M KOH | Co-CNTs-MW | 200 | / | 95.4 | 54.1 |  | [9] |
|  | 0.1 M KHCO_3_ |  | 100 | 60 | 85.6 | 39.5 |  |  |
|  | KHCO_3_ | CoPc@CNT0.5M | 100 | 13 | 96.2 | 35 |  | [10] |
|  | KOH | a cobalt-tetrapyridino-porphyrazine complex supported on carbon black together with potassium | 100 | 168 | (95.0 ± 1.4) | 68 |  | [11] |
|  | 0.1 M KOH | Ag GDE | 200 | - | 99 | 47.5 |  | [12] |
|  | 0.1 M H_2_SO_4_ | c-PDDA decorated Ag NPs | 200 | 36 | 90 | - |  | [13] |
|  | 0.1 M KOH | Pd/C | 200 | - | 98 | 48.8 |  | [14] |
|  |  | Cu | 350 | - | 81.9 | 30.5 |  |  |
|  |  | Pd/C | 100 | 40 | 98 | 46.6-50.1 |  |  |
|  | 2 M KOH | MWNT/PyPBI/Au | 99 | 8 | 86-90 | 63.8 |  | [15] |
|  |  |  | 158 | - | - | 49.4 |  |  |
|  | 0.1 M KHCO_3_ | Ni single atom | 400 | - | 92.5 | 35.2 |  | [16] |
|  |  |  | 100 | 40 | 97 | 43 |  |  |
| **SOEC** | YSZ | Ni-YSZ | 815 | 55 | 95 | 89 | 800 | [17] |
|  | SSZ | LSFNbx-GDC | 500 | 40 | - | <76 | 800 | [18] |
|  | LGSM | SFRuM-GDC | 450 | 567 | 97 | 77.6 | 800 | [19] |
|  | LGSM | HE-PSCFMMN-CFA@FeO | 750 | 200 | 98.9 | 76.64 | 800 | [20] |
|  | YSZ | 12.8 GDC-SFM | 446 | 55 | 96 | 55.8 | 800 | [21] |
| **Ion liquids** | 0.1mol·L−1 [Bmim][OTf] + 0.1 mol·L−1TBAPF6 in MeCN | Bi film | 15±2 | 3 | 88±5 | <39.9 | - | [22] |
|  |  | Sn film | 12±1 |  | 87±4 | <39.4 |  |  |
|  |  | Pb film | 4 ± 1 |  | 75 ± 3 | <34.0 |  |  |
|  |  | Bi22Sn50Pb28 | 4.5 ± 0.5 |  | 85 ± 4 | <38.5 |  |  |
|  | [BMIM]PF6 | BI-CMEC | 31±12 | 0.6 | 82±12 | <43.9 | - | [23] |
|  | 0.1 mol·L−1 [Bmim][OTf] + 0.1 mol·L−1TBAPF6 in MeCN | Cu6Sn5 | 3.9±0.2 | 2.5 | 34.4 ± 2.7 | <45.24 | 200 | [24] |

**Abbreviation Index**

**NP**: nanoparticle **GDL**: gas diffusion layer **GDE**: gas diffusion electrode **YSZ**: Yttria-Stabilized Zirconia

**SSZ**: Scandium-Stabilized Zirconia **LSFNbx-GDC**: La_0.6_Sr_0.4_Fe_0.9_Nb_0.1_O_3-δ_-Gd_0.1_Ce_0.9_O_2-δ_ **LGSM**: La_0.8_Sr_0.2_-Ga_0.8_Mg_0.2_O_3−δ_

**MWNT/PyPBI/Au**: Au nanoparticlessupported on poly(2,2’-(2,6-pyridine)-5,5’-bibenzimidazole)polymer (PyPBI) wrapped multiwall carbon nanotubes(MWNTs)

**SFRuM-GDC**: SFRuM perovskite phase and Gd0.2Ce0.8O1.9 (GDC) fluorite phase were used as the composite cathode (SFRuM-GDC)

**HE-PSCFMMN-CFA@FeO**: high-entropy Pr_0.8_Sr_1.2_(CuFe)_0.4_Mo_0.2_Mn_0.2_Nb_0.2_O_4-δ_ (HE-PSCFMMN) layered perovskite uniformly coated with in situ exsolved core-shell structured CuFe alloy@FeOx (CFA@FeO) nanoparticles

**GDC-SFM**: Gd_0.2_Ce_0.8_O_1.9_-Sr_2_Fe_1.5_Mo_0.5_O_6-δ_

**[Bmim]**: 1-Butyl-3-methylimidazolium **[OTf]**: Trifluoromethanesulfonate  **TBAPF6**: Tetrabutylammonium hexafluorophosphate

**MeCN**: Acetonitrile

**Table S3.** The comparative analysis among different CO_2_ electrolysis systems for producing CO

| Electrolytes | Cathodic potential  (V *vs.* Li^+^/Li) | Cathodic overpotential^a^  (mV) | Cathodic current density (mA cm^-2^) | CO_2_ER potential (V *vs*. NHE) | OER potential (V *vs*. NHE) | Reference |
| --- | --- | --- | --- | --- | --- | --- |
| LNK-CO_3_-0.06Na_2_B_4_O_7_ | 1.342 | 168 | 100 | -0.748 | 0.505 | This work |
|  | 1.293 | 224 | 200 | -0.797 | 0.638 |  |
|  | 1.150 | 373 | 400 | -0.940 | 0.772 |  |
|  | 1.061 | 455 | 500 | -1.029 | 0.833 |  |
| 0.5 M KHCO_3_ | - | - | 100 | -1.291 |  | [25] |
| 0.1 M KHCO_3_ | - | - | 200 | -1.203 |  | [26] |
| 0.5 M KOH | - | - | 276 | -1.650 |  | [27] |
| 0.5 M KHCO_3_ | - | - | 452 | -1.410 |  | [5] |

^a^Theoretical potential of cathodic reaction “CO_2_ + 2e = CO (g) + O^2-^” is 1.51 V (*vs.* Li^+^/Li) and -0.580 V (*vs.* NHE), respectively.

**Table S4**. Comparison of cost expense among typical CO_2_ electrolysis system

|  | **Electrolytes** | **Electrolyte cost ($/kg)** | **Electrode** | **Electrode cost ($/kg)** | **Reference** |
| --- | --- | --- | --- | --- | --- |
| **Molten salt** | LNK-0.06Na_2_B_4_O_7_ | 125 | Cu | 37 | This work |
| **Aqueous electrolyte** | KOH | 5-10 | Au/GDL | 80000-90000 | [7] |
|  | KHCO_3_ | 5-10 | Ag/GDL | 2000-3000 | [8] |
| **SOEC** | YSZ | 500-600 | Ni-YSZ | 100-150 | [17] |
|  | SSZ | 100-500 | LSFNbx-GDC | 200-800 | [18] |

**References:**

[1] I. A. Sysoev, V. A. Ershov, V. V. Kondrat’ev, *Metallurgist* **2015**, *59*, 518–525.

[2] Z. Sun, C. Liu, G. Lu, X. Song, S. Sun, Y. Sun, J. Yu, *Energy Fuels* **2011**, *25*, 2655–2663.

[3] V. Kaplan, E. Wachtel, K. Gartsman, Y. Feldman, I. Lubomirsky, *J. Electrochem. Soc.* **2010**, *157*, B552.

[4] F. Matsuura, T. Wakamatsu, S. Natsui, T. Kikuchi, R. O. Suzuki, *ISIJ Int.* **2015**, *55*, 404–408.

[5] J. Li, H. Zeng, X. Dong, Y. Ding, S. Hu, R. Zhang, Y. Dai, P. Cui, Z. Xiao, D. Zhao, L. Zhou, T. Zheng, J. Xiao, J. Zeng, C. Xia, *Nat Commun* **2023**, *14*, 340.

[6] J. Yin, J. Jin, Z. Yin, L. Zhu, X. Du, Y. Peng, P. Xi, C.-H. Yan, S. Sun, *Nat Commun* **2023**, *14*, 1724.

[7] M. C. O. Monteiro, M. F. Philips, K. J. P. Schouten, M. T. M. Koper, *Nat Commun* **2021**, *12*, 4943.

[8] X. Wang, P. Li, J. Tam, J. Y. Howe, C. P. O’Brien, A. Sedighian Rasouli, R. K. Miao, Y. Liu, A. Ozden, K. Xie, J. Wu, D. Sinton, E. H. Sargent, *Nat Sustain* **2024**, *7*, 931–937.

[9] J. W. Sun, X. Wu, P. F. Liu, J. Chen, Y. Liu, Z. X. Lou, J. Y. Zhao, H. Y. Yuan, A. Chen, X. L. Wang, M. Zhu, S. Dai, H. G. Yang, *Nat Commun* **2023**, *14*, 1599.

[10] M. Shen, L. Ji, D. Cheng, Z. Wang, Q. Xue, S. Feng, Y. Luo, S. Chen, J. Wang, H. Zheng, X. Wang, P. Sautet, J. Zhu, *Joule* **2024**, *8*, 1999–2015.

[11] S. Sato, K. Sekizawa, S. Shirai, N. Sakamoto, T. Morikawa, *Science Advances* **2023**, *9*, eadh9986.

[12] J. Disch, L. Bohn, S. Koch, M. Schulz, Y. Han, A. Tengattini, L. Helfen, M. Breitwieser, S. Vierrath, *Nat Commun* **2022**, *13*, 6099.

[13] H.-G. Qin, Y.-F. Du, Y.-Y. Bai, F.-Z. Li, X. Yue, H. Wang, J.-Z. Peng, J. Gu, *Nat Commun* **2023**, *14*, 5640.

[14] P. Wei, H. Li, L. Lin, D. Gao, X. Zhang, H. Gong, G. Qing, R. Cai, G. Wang, X. Bao, *Sci. China Chem.* **2020**, *63*, 1711–1715.

[15] S. Verma, Y. Hamasaki, C. Kim, W. Huang, S. Lu, H.-R. M. Jhong, A. A. Gewirth, T. Fujigaya, N. Nakashima, P. J. A. Kenis, *ACS Energy Lett.* **2018**, *3*, 193–198.

[16] Z.-Y. Wu, P. Zhu, D. A. Cullen, Y. Hu, Q.-Q. Yan, S.-C. Shen, F.-Y. Chen, H. Yu, M. Shakouri, J. D. Arregui-Mena, A. Ziabari, A. R. Paterson, H.-W. Liang, H. Wang, *Nat. Synth* **2022**, *1*, 658–667.

[17] A. Ozden, Y. Wang, F. Li, M. Luo, J. Sisler, A. Thevenon, A. Rosas-Hernández, T. Burdyny, Y. Lum, H. Yadegari, T. Agapie, J. C. Peters, E. H. Sargent, D. Sinton, *Joule* **2021**, *5*, 706–719.

[18] S. Wang, B. Qian, Z. Wang, B. Yin, Y. Zheng, L. Ge, H. Chen, H. Yang, *Journal of Alloys and Compounds* **2021**, *888*, 161573.

[19] H. Lv, L. Lin, X. Zhang, R. Li, Y. Song, H. Matsumoto, N. Ta, C. Zeng, Q. Fu, G. Wang, X. Bao, *Nat Commun* **2021**, *12*, 5665.

[20] Z. Wang, T. Tan, K. Du, Q. Zhang, M. Liu, C. Yang, *Advanced Materials* **2024**, *36*, 2312119.

[21] H. Lv, Y. Zhou, X. Zhang, Y. Song, Q. Liu, G. Wang, X. Bao, *Journal of Energy Chemistry* **2019**, *35*, 71–78.

[22] T. Kunene, A. Atifi, J. Rosenthal, *ACS Appl. Energy Mater.* **2020**, *3*, 4193–4200.

[23] J. Medina-Ramos, J. L. DiMeglio, J. Rosenthal, *J. Am. Chem. Soc.* **2014**, *136*, 8361–8367.

[24] R. L. Sacci, S. Velardo, L. Xiong, D. A. Lutterman, J. Rosenthal, *Energies* **2019**, *12*, 3132.

[25] T. Möller, W. Ju, A. Bagger, X. Wang, F. Luo, T. N. Thanh, A. S. Varela, J. Rossmeisl, P. Strasser, *Energy Environ. Sci*. **2019**, 12, 640–647.

[26] T. Zheng, K. Jiang, N. Ta, Y. Hu, J. Zeng, J. Liu, H. Wang, *Joule* **2019**, 3, 265–278.

[27] L. Lin, H. Li, C. Yan, H. Li, R. Si, M. Li, J. Xiao, G. Wang, X. Bao, *Adv. Mater*. **2019**, 31, 1903470.
